# Rapid increase in the prevalence of metronidazole-resistant Helicobacter pylori in the Netherlands.

**DOI:** 10.3201/eid0303.970320

**Published:** 1997

**Authors:** E J van der Wouden, A A van Zwet, G D Vosmaer, J A Oom, A de Jong, J H Kleibeuker

**Affiliations:** Bethesda Hospital, Hoogeveen, The Netherlands.

## Abstract

The prevalence of primary metronidazole resistance of Helicobacter pylori was studied in one Dutch hospital from 1993 to 1996 and in two additional Dutch hospitals in 1993 and 1996. All cultures of antral biopsy specimens yielding H. pylori in the study period were evaluated, except those from patients who had received anti-H. pylori treatment; 1,037 H. pylori strains, all from different patients were included. Metronidazole resistance was determined by disk diffusion in 1993 and by Epilipsometer-test in 1994 to 1996. Metronidazole resistance increased from 7% (18/245) in 1993 to 32% (161/509) in 1996. More patients with nonulcer dyspepsia and more non-Western European patients were seen in 1996 than in 1993, but age and sex differences were not observed. A comparable increase in metronidazole resistance was observed in both nonulcer dyspepsia patients and peptic ulcer patients, and the prevalence of metronidazole resistance in Western Europeans increased from 5% in 1993 to 28% in 1996.


### Rapid Increase in the Prevalence of Metronidazole-Resistant *Helicobacter pylori* in the Netherlands

The prevalence of primary metronidazole resistance of *Helicobacter pylori* was studied in one Dutch hospital from 1993 to 1996 and in two additional Dutch hospitals in 1993 and 1996. All cultures of antral biopsy specimens yielding *H. pylori* in the study period were evaluated, except those from patients who had received anti-*H. pylori* treatment; 1,037 *H. pylori* strains, all from different patients were included. Metronidazole resistance was determined by disk diffusion in 1993 and by Epilipsometer-test in 1994 to 1996. Metronidazole resistance increased from 7% (18/245) in 1993 to 32% (161/509) in 1996. More patients with nonulcer dyspepsia and more non-Western European patients were seen in 1996 than in 1993, but age and sex differences were not observed. A comparable increase in metronidazole resistance was observed in both nonulcer dyspepsia patients and peptic ulcer patients, and the prevalence of metronidazole resistance in Western Europeans increased from 5% in 1993 to 28% in 1996.

Since the first description (1) of *Helicobacter pylori* and the acceptance of its role in the pathogenesis of peptic ulcer disease (PUD) (2), different regimens to eradicate this microorganism have been used in clinical practice (3). Metronidazole has frequently been used as a component in these treatment regimens. *H. pylori* resistance to metronidazole has been associated with treatment failure (4-7). Recently, an increase of metronidazole resistance has been reported from different parts of the world (8-13). This retrospective study describes the prevalence of primary metronidazole resistance occurring in *H. pylori* strains in 1993 and 1996 in three regional hospitals in the northern part of the Netherlands and in 1994-95 in one of these hospitals.

### Sampling

All cultures of antral biopsy specimens yielding growth of *H. pylori* in the study period were considered for evaluation. Previous anti-*H. pylori* treatment was the only reason for exclusion. All 1,037 *H. pylori* strains evaluated were isolated from different patients. Biopsy specimens for culture were taken within 3 cm of the pylorus. Endoscopes and biopsy equipment were thoroughly cleaned with a detergent and disinfected with 2% glutaraldehyde in an automatic washing machine between procedures. Culture was performed as described elsewhere (14).

### Susceptibility Testing

Susceptibility to metronidazole was determined by disk diffusion in 1993 and the Epilipsometer-test (E-test) in 1994-1996. For these tests, plates were injected with a suspension adjusted to a turbidity approximating that of a McFarland No. 3 standard (15). For disk diffusion, a 5-μg disk (Mast Laboratories, Liverpool, United Kingdom) was used and read after at least 3 days of incubation. Strains with an inhibition zone of 10 mm or more were regarded as susceptible (16). The E-test (AB Biodisk, Solna, Sweden)(17) was performed according to the instructions of the manufacturer and read after at least 3 days. The strains were considered metronidazole resistant when the minimum inhibitory concentration was above 8  g/ml (18).

To test the equivalence of the two methods of susceptibility testing, a prospective study compared the E-test and disk diffusion. In 124 different *H. pylori* strains, results were concurrent in all but six.

### Prevalence of Metronidazole-Resistant *H. pylori*

In one of the hospitals (Hospital C), data were available on endoscopic diagnosis, age, and ethnic background of the patients from whom the strains were isolated in 1993 and 1996. In this hospital, it was possible to compare the prevalence of metronidazole resistance in PUD patients with that in nonulcer dyspepsia (NUD) patients and to look at resistance rates in patients of different ethnic backgrounds. Statistical analysis was performed by Fisher's exact test on binomial data and Student's t test on continuous data. Differences were considered significant when $p < 0.05$.

The number of *H. pylori* strains isolated in the three hospitals was 245 in 1993 and 509 in 1996. In Hospital C, an additional 137 strains from 1994 and 146 strains from 1995 were studied. Looking at the sex of the study population, we found a male-to-female ratio in 1993 of 1.75:1, in 1994 of 1.36:1, in 1995 of 1:1, and in 1996 of 1.18:1 (the total male-to-female ratio was 1.28:1). The proportion of women in the population examined was higher in 1996 (46%) than in 1993 (36%)($p = 0.02$). The prevalence of metronidazole resistance did not differ, however, between men and women. The prevalence of metronidazole resistance increased significantly from 1993 to 1996 in the total sample group (Figure 1) and also among men (from 7% to 30%, $p < 0.0001$) and women (from 8% to 33%, $p < 0.0001$).

In Hospital C, the number of strains isolated from PUD patients decreased from 83% to 38%. No significant difference, however, was noted in the prevalence of resistance between NUD patients and PUD patients in 1993 or in 1996 (Figure 2, $p < 0.0001$). In Hospital C more strains from non-Western European patients were included in 1996 than in 1993 ($p = 0.04$), and the prevalence of metronidazole resistance was higher in this group than in the total population. Exclusion of this patient group still resulted in an increase in the prevalence among the Western Europeans ($p < 0.0001$). The mean age of the patients from whom *H. pylori* strains were isolated in Hospital C was the same in 1993 and 1996 (55 ± 14 [mean ± standard deviation] and 54 ± 16 years, respectively.)

Our study shows a rapidly increasing prevalence of metronidazole resistance in *H. pylori* in the Netherlands. This increase was observed in all three hospitals included in the study. Our results are consistent with the findings of some investigators (8-13) but not with those of others (19,20). It confirms our own previous experience of increasing resistance in this part of the Netherlands (21,22).

We explored the possibility that the observed rise in metronidazole resistance was due to some known confounding factor such as age, sex, endoscopic diagnosis, or ethnicity. More *H. pylori* strains were isolated from NUD patients in 1996 than in 1993. In 1996, the proportions of women and foreigners in the examined populations were also higher. In contrast with the results of Ching et al. (23), however, we found that the prevalence of metronidazole resistance in NUD patients and in PUD patients was the same. Furthermore, the prevalence of metronidazole resistance was comparable among men and women in both 1993 and 1996. Exclusion of the non-Western European patients from the analysis still showed a rapid increase in metronidazole resistance. Several authors have suggested that the prevalence of metronidazole resistance is higher in the young and middle-aged (19,20,24-26). In our study population, however, the mean age was the same in 1993 and 1996.

The use of different techniques to measure metronidazole susceptibility could confound the validity

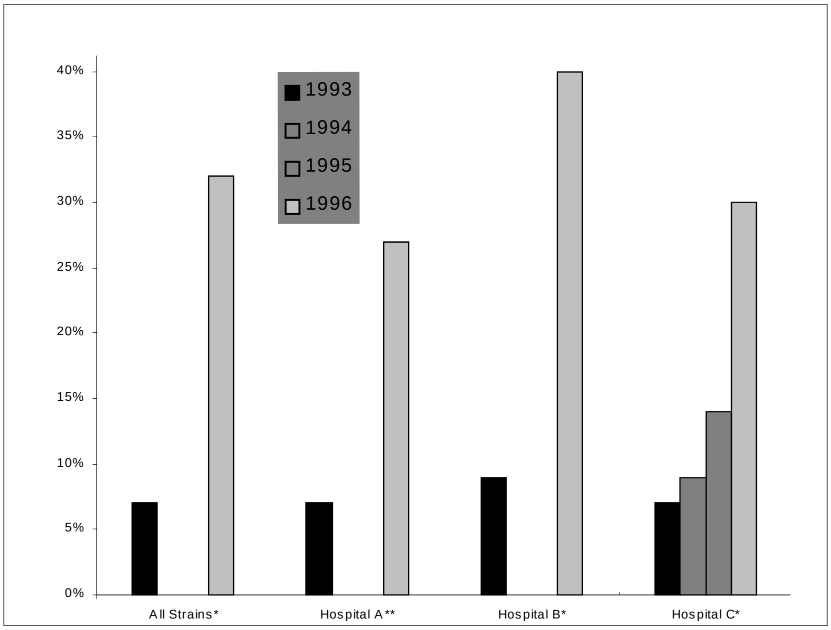

Figure 1. The increase in prevalence of metronidazole resistance of *H. pylori* from 1993 to 1996 in three different hospitals. Data presented as percent of strains that were resistant.
*p< 0.0001 1993 vs. 1996
**p<0.001 1993 vs. 1996

of our results (26). However, our prospective study comparing the E-test and disk diffusion, as well as other studies (27,28), show a very high intertest agreement when using the above-stated criteria for metronidazole resistance. We cannot exclude the possibility that other methodologic factors are involved. However, because procedures were standardized and the increase was observed in three different hospitals, each with its own laboratory, we consider this unlikely. Therefore, the observed rapid increase seems real and is relevant for clinical practice (4-7).

### Possible Causes of Resistance

Several authors have suggested that the use of imidazoles for other indications, such as gynecologic infections, could account for the resistance increase (6,22-24,28,29). This would also explain the higher prevalence of metronidazole resistance in women that has been observed in several studies (6,19,21,25). Our study, however, did not show a significant difference between men and women or a more apparent increase in women. Moreover, out-of-hospital prescription of metronidazole in the Netherlands increased only slightly from 1989 until 1995 (Figure 3). Some authors have suggested that imidazole-containing regimens themselves could be the cause (25,29,30). However, we consider this unlikely. First, we excluded all strains that were isolated after known anti-*H. pylori* treatment. We cannot completely exclude the possibility that some of the patients had been treated by their general practitioner without our knowledge. We are, however, confident that this is a rare occurrence because in our region most physicians prescribe their treatment on the basis of endoscopic findings and culture of the biopsy specimens, and we purposely excluded all patients from whom *H. pylori* was previously isolated. In our region, breath testing is not available for general practitioners, and serologic tests are rarely used. Moreover, imidazole-containing anti-*H. pylori* regimens are highly effective (3,7), and metronidazole resistance could be induced only in the few *H. pylori* strains escaping eradication. Finally, as infection is rare during adulthood, it is unlikely that strains rendered resistant in that way spread in the population (31,32). Therefore, although general practitioners may have been treating *H. pylori* infections more frequently in recent years, it seems unlikely that this could have caused the observed fourfold increase in the prevalence of resistance.

The cause of the rapid increase in metronidazole resistance in *H. pylori* that we observed can only be a matter of speculation. Apparently, metronidazole-resistant *H. pylori* strains somehow have a survival advantage, and the increase in metronidazole resistance may be the result of some as yet unknown environmental pressure. Our study suggests that the prevalence of

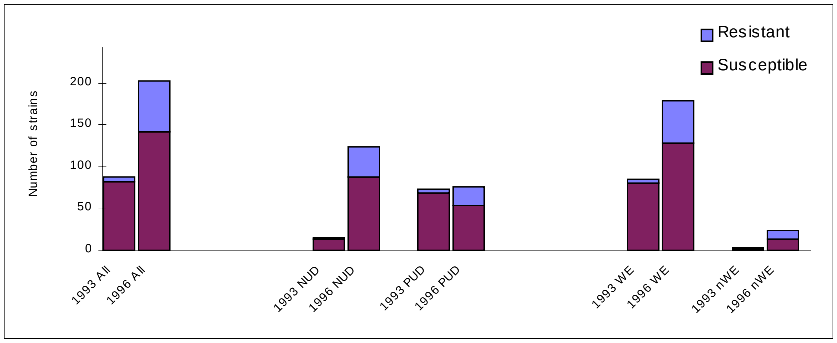

Figure 2. Distribution of metronidazole-resistant strains of *H. pylori* in 1993 and 1996 in Hospital C. All = total number, NUD = Nonulcer dyspepsia patients, PUD = peptic ulcer disease patients, nWE = non-Western Europeans.

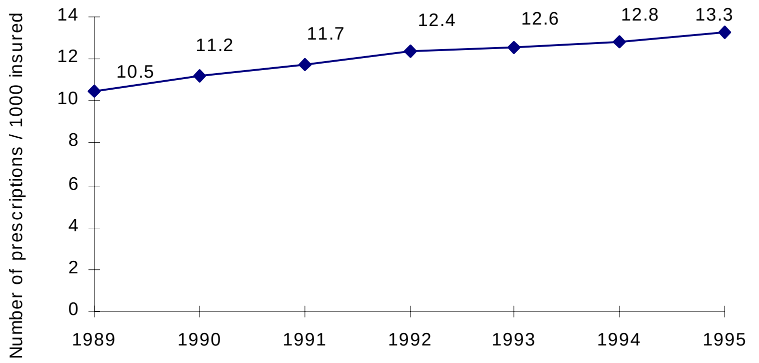

Figure 3. The out-of-hospital use of metronidazole in the Netherlands in the years 1989 to 1995. Data derived from the Health Insurance Council (Ziekenfondsraad). Drug Information Project, Amstelveen, the Netherlands.

metronidazole resistance in *H. pylori* is rapidly increasing in the Netherlands. The cause of this increase, however, is still elusive.

**E.J. van der Wouden,* A.A. van Zwet,† J.C. Thijs,* G.D.C. Vosmaer,‡ J.A.J. Oom,§ A. de Jong,† and J.H. Kleibeuker¶**
*Bethesda Hospital, Hoogeveen, The Netherlands; †Regional Public Health Laboratory Groningen/ Drenthe, Hoogeveen, The Netherlands; ‡Scheper Hospital, Emmen, The Netherlands; §Diaconessenhuis, Meppel, The Netherlands; and ¶University Hospital, Groningen, The Netherlands

### References

1. Warren JR. Unidentified curved bacilli on gastric epithelium in active chronic gastritis. Lancet 1983;i:1273.
2. National Institutes of Health. Consensus conference.*Helicobacter pylori* in peptic ulcer disease. JAMA 1994;272:65-9.
3. Van der Hulst RWM, Keller JJ, Rauws EAJ, Tytgat GNJ. Treatment of *Helicobacter pylori* infection: review of the world literature. Helicobacter 1996;1:6-19.
4. Van Zwet AA, Thijs JC, Oom JAJ, Hoogeveen J, Düringshoff BL. Failure to eradicate *Helicobacter pylori* in patients with metronidazole resistant strains. Eur J Gastroenterol Hepatol 1993;5:185-6.
5. Bell GD, Powell K, Burridge SM, Pallecaros A, Jones PH, Gant PW, et al. Experience with “triple” anti-*Helicobacter pylori* eradication therapy: side effects and the importance of testing the pretreatment bacterial isolate for metronidazole resistance. Aliment Pharmacol Ther 1992;6:427-35.
6. Rautelin H, Seppäla K, Renkonen OV, Vainio U, Kosunen TU. Role of metronidazole resistance in therapy of *Helicobacter pylori* infections. Antimicrob Agents Chemother 1992;36:163-6.
7. Thijs JC, van Zwet AA, Thijs WJ, Van der Wouden EJ, Kooy A. One week triple therapy with omeprazole, amoxicillin, and tinidazole for *Helicobacter pylori* infection: the significance of imidazole susceptibility. Aliment Pharmacol Ther 1997;11:305-9.
8. Reddy R, Osato M, Gutiérrez O, Kim JG, Graham DY. Metronidazole resistance is high in Korea and Colombia and appears to be rapidly increasing in the U.S [abstract]. Gastroenterology 1996;110:A238.
9. Ling TWK, Cheng AFB, Sung JJY, Yiu PYL, Chung SSC. An increase in *Helicobacter pylori* strains resistant to metronidazole: a five year study. Helicobacter 1996;1:57-61.
10. Xia HX, Keane CT, O’Morain CA. A 5-year survey of metronidazole and claritromycin resistance in clinical isolates of *Helicobacter pylori* [abstract]. Gut 1996;39:A6.
11. Lopez-Brea M, Martinez MJ, Domingo D, Sanchez Romero I, Sanz JC, Alarcon T. Evolution of the resistance to several antibiotics in *Helicobacter pylori* over a four year period [abstract]. Gut 1995;37:A97.
12. Teo EK, Fock KM, Ng TM, Chia SC, Khor CJL, Tan AL, et al. Primary and secondary metronidazole resistant *Helicobacter pylori* in an urban asian population [abstract]. Gut 1996;39:A23-4.
13. Weissfeld AS, Simmons DE, Vance PH, Trevino E, Kidd S, Greski-Rose P. In vitro susceptibility of pretreatment isolates of *Helicobacter pylori* from two multicenter United States clinical trials [abstract]. Gastroenterology 1996;110:A295.
14. Van Zwet AA, Thijs JC, Roosendaal R, Kuipers EJ, Pena S, de Graaff J. Practical diagnosis of *Helicobacter pylori* infection. Eur J Gastroenterol Hepatol 1996;8:501-7.
15. Berger SA, Gorea A, Moskowitz M, Santo M, Gilat T. Effect of inoculum size on antimicrobial susceptibility of *Helicobacter pylori*. Eur J Clin Microbiol Infect Dis 1993;12:782-3.
16. DeCross AJ, Marshall BJ, McCallum RW, Hoffman SR, Barrett LJ, Guerrant RL. Metronidazole susceptibility testing for *H. pylori*: comparison of disk, broth and agar dilution methods and their clinical relevance. J Clin Microbiol 1993;31:1971-4.
17. Graham DY, Börsch GM. The who’s and when’s of therapy for *Helicobacter pylori* [editorial]. Am J Gastroenterol 1990;85:1552-5.
18. National Committee for Clinical Laboratory Standards. Standard MF-A. Villanova, PA: National Committee for Clinical Laboratory Standards; 1990.
19. Karim QN, Logan RPH. *Helicobacter pylori (H. pylori)* antimicrobial resistance in the UK [abstract]. Gut 1996;39:A15.
20. De Koster E, Cozzoli A, Jonas C, Ntounda R, Butzler JP, Deltenre M. Six years resistance of *Helicobacter pylori* to macrolides and imidazoles [abstract]. Gut 1996;39:A5.
21. Thijs JC, Van Zwet AA, Oey HB. Efficacy and side effects of a triple drug regimen for eradication of *Helicobacter pylori*. Scand J Gastroenterol 1993;28:934-8.
22. Van Zwet AA, De Boer WA, Schneeberger PM, Weel J, Jansz AR, Thijs JC. Prevalence of primary *Helicobacter pylori* resistance to metronidazole and claritromycin in The Netherlands. Eur J Clin Microbiol Infect Dis 1996:15:861-4.
23. Ching CK, Leung KP, Yung RWH, Lam SK, Wong BC, Lai KC, Lai CL. Prevalence of metronidazole resistant *Helicobacter pylori* strains among Chinese peptic ulcer disease patients and normal controls in Hong Kong. Gut 1996;38:675-8.
24. Banatvala N, Davies GR, Abdi Y, Clements L, Rampton DS, Hardie JM, et al. High prevalence of *Helicobacter pylori* metronidazole resistance in migrants to east London: relation with previous nitroimidazole exposure and gastroduodenal disease. Gut 1994;35:1562-6.
25. Glupczynski Y, Burette A, De Koster E, Nyst JF, Deltenre M, Cadranel S, et al. Metronidazole resistance in *Helicobacter pylori* [letter]. Lancet 1990;335:976-7.
26. European Study Group on Antibiotic Susceptibility of *Helicobacter pylori*. Results of a multicentre European survey in 1991 of metronidazole resistance in *Helicobacter pylori*. Eur J Clin Microbiol Infect Dis 1992;11:777-81.

27. Hirschl AM, Hirschl MM, Rotter ML. Comparison of three methods for the determination of the sensitivity of *Helicobacter pylori* to metronidazole. J Antimicrob Chemother 1993;32:45-9.
28. Midolo PD, Turnidge J, Lambert JR, Bell JM. Validation of a modified Kirby-Bauer disk diffusion method for metronidazole susceptibility testing of *Helicobacter Pylori*. Diagn Microbial Infect Dis 1995;21:135-40.
29. Becx MCJM, Janssen AJHM, Clasener HAL, de Koning RW. Metronidazole-resistant *Helicobacter pylori* [letter]. Lancet 1990;335:539-40.
30. Weil J, Bell GD, Powell K, Jobson R, Trowell JE, Gant P, Jones PH. *Helicobacter pylori* and metronidazole resistance [letter]. Lancet 1990;336:1445.
31. Walt RP. Metronidazole resistant *H. pylori*—of questionable clinical importance. Lancet 1996;348:489-90.
32. Megraud F. Epidemiology of *Helicobacter pylori* infection: where are we in 1995? Eur J Gastroenterol Hepatol 1995;7:292-5.
